# *Bradyrhizobium diazoefficiens* USDA 110 displays plasticity in the attachment phenotype when grown in different soybean root exudate compounds

**DOI:** 10.3389/fmicb.2023.1190396

**Published:** 2023-05-18

**Authors:** Armaan Kaur Sandhu, McKenzie Rae Brown, Senthil Subramanian, Volker S. Brözel

**Affiliations:** ^1^Departments of Biology and Microbiology, South Dakota State University, Brookings, SD, United States; ^2^Department of Agronomy, Horticulture and Plant Science, South Dakota State University, Brookings, SD, United States; ^3^Department of Biochemistry, Genetics and Microbiology, University of Pretoria, Pretoria, South Africa

**Keywords:** *Bradyrhizobium diazoefficiens*, soybean, root, attachment, biofilm, phenotype, root exudate, phenotypic plasticity

## Abstract

**Introduction:**

*Bradyrhizobium diazoefficiens*, a symbiotic nitrogen fixer for soybean, forms nodules after developing a symbiotic association with the root. For this association, bacteria need to move toward and attach to the root. These steps are mediated by the surface and phenotypic cell properties of bacteria and secreted root exudate compounds. Immense work has been carried out on nodule formation and nitrogen fixation, but little is known about the phenotype of these microorganisms under the influence of different root exudate chemical compounds (RECCs) or how this phenotype impacts the root attachment ability.

**Methods:**

To address this knowledge gap, we studied the impact of 12 different RECCs, one commonly used carbon source, and soil-extracted solubilized organic matter (SESOM) on attachment and attachment-related properties of *B. diazoefficiens* USDA110. We measured motility-related properties (swimming, swarming, chemotaxis, and flagellar expression), attachment-related properties (surface hydrophobicity, biofilm formation, and attachment to cellulose and soybean roots), and surface polysaccharide properties (colony morphology, exopolysaccharide quantification, lectin binding profile, and lipopolysaccharide profiling).

**Results and discussion:**

We found that USDA 110 displays a high degree of surface phenotypic plasticity when grown on the various individual RECCs. Some of the RECCs played specific roles in modulating the motility and root attachment processes. Serine increased cell surface hydrophobicity and root and cellulose attachment, with no EPS formed. Gluconate and lactate increased EPS production and biofilm formation, while decreasing hydrophobicity and root attachment, and raffinose and gentisate promoted motility and chemotaxis. The results also indicated that the biofilm formation trait on hydrophilic surfaces (polystyrene) cannot be related to the attachment ability of *Bradyrhizobium* to the soybean root. Among the tested phenotypic properties, bacterial cell surface hydrophobicity was the one with a significant impact on root attachment ability. We conclude that USDA 110 displays surface plasticity properties and attachment phenotype determined by individual RECCs from the soybean. Conclusions made based on its behavior in standard carbon sources, such as arabinose or mannitol, do not hold for its behavior in soil.

## Introduction

**Figure 1:**
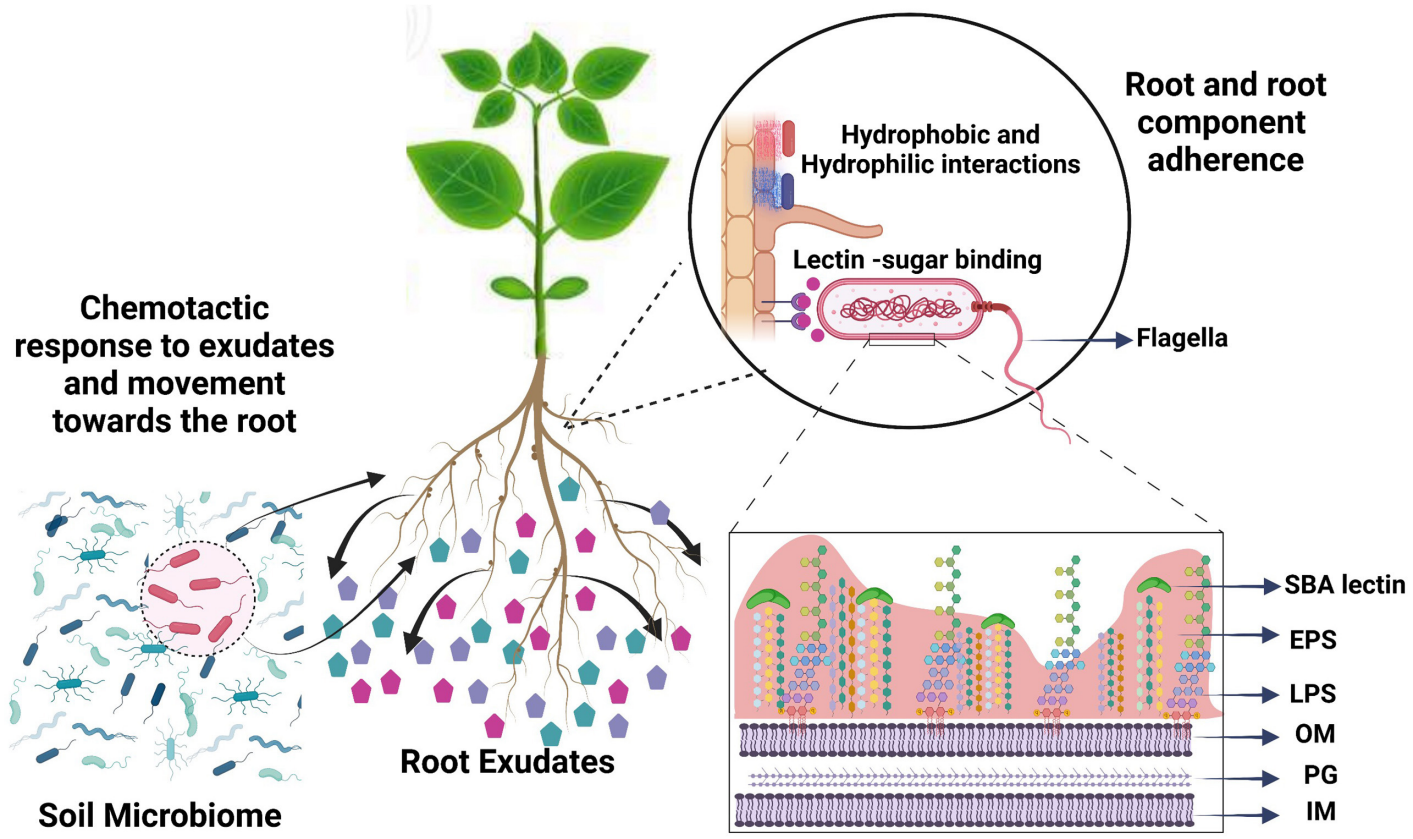
Root colonization is a multiphase process involving the translocation of bacteria from soil to the root and adherence to the root surface. Bacterial cells are chemotactically attracted by root exudate compounds and use their flagella to drive root-directed movement. For root adherence, bacteria use hydrophobic interactions and surface sugars. Subsequently, microcolony formation is moderated by extracellular polymeric substances. We compared phenotypic traits that play role in root attachment under the influence of soybean root exudate compounds, to understand the role of each in root attachment. SBA, soybean agglutinin lectin; EPS, extracellular polymeric substances; LPS, lipopolysaccharides; OM, outer membrane; PG, peptidoglycan; IM, inner membrane.

The symbiotic relationship of *Bradyrhizobium* with the host legume soybean (*Glycine max*) involves the attachment of bacteria to the root hair, followed by root colonization, infection thread, and nodule formation (Mendoza-Suárez et al., 2021). Root attachment, the prerequisite for successful nodulation, is a multistep process and is influenced by both bacterial and plant root phenotypic properties and activities. One of these root activities is the secretion of photosynthetically fixed carbon, which acts as a chemoattractant or repellent by developing a chemical gradient, and as a carbon source for bacteria in the rhizosphere (Figure 1) (Hayat et al., 2017). Exudates sensed as chemoattractants cause bacteria to swim or swarm toward the root surface, even overcoming electrostatic repulsion from the root surface (Vesper and Bauer, 1986; Knights et al., 2021). Flagella also help the bacteria to migrate across the root surface in later attachment stages (Knights et al., 2021). Once at the root, rhizobia develop weak reversible binding known as primary attachment through hydrophobic and electrostatic forces and surface sugar-based soybean lectin binding (Rodríguez-Navarro et al., 2007). For secondary, irreversible attachment, rhizobia-produced cellulose fibrils and extracellular polysaccharides play fundamental roles. Irreversible attachment leads to the formation of microcolonies of rhizobia on the surface of the root, which grow to form biofilms with the support of root exudate chemical compounds (RECCs) as a carbon source (Downie, 2010). Root colonization is followed by root hair deformation and curling which ends up entrapping bacteria and leads to infection thread formation (Tsyganova et al., 2021). Each of these bacterial surface properties, including flagella, pili, cell surface hydrophobicity, surface sugar moieties, and extracellular polysaccharides, have their own specific contribution in establishing successful rhizobia–legume interaction required for nodule formation (Figure 1). However, the exact role and significance of each of these bacterial surface properties in attachment are unknown.

Hydrophobic domains at the root surface have been implicated in bacterial attachment, but we have not been able to find much detail on the nature of these domains (Wheatley and Poole, 2018; Knights et al., 2021). The soybean root epidermis, the outermost layer, which is in direct contact with the soil environment, predominantly contains cellulose microfibrils, matrix material, and water. The matrix material is likely suberin, a heteropolymer of phenolics and wax components, but little is known about its specific structure and distribution with the cellulose at the root surface of soybean (Fang, 2006). As the major component of the root hair surface, cellulose is likely one of the predominant compounds encountered by rhizobia. Whether cellulose microfibrils play a role in rhizobia root attachment remains unknown. Along with the plant–microbe interaction, RECCs can also influence bacterial phenotype. Numerous studies have demonstrated that root exudate cocktails can induce the expression of certain genes in rhizospheric bacteria (Sasse et al., 2018). The transcriptome responses of rhizosphere *Pseudomonas, Rhizobium phaseoli*, and *B. diazoefficiens* to exudates of *Brachypodium*, maize and common bean, and soybean, respectively, have revealed differential expression of bacterial surface property-related genes (Liu et al., 2017; Aguirre-Noyola et al., 2021; Mavrodi et al., 2021). These gene expressions have been believed to increase the nodulation competitiveness of *Bradyrhizobium* strains (Liu et al., 2015, 2017; Mavrodi et al., 2021). Peanut root exudates enhanced chemotaxis and biofilm formation by *Rhizobium* and common bean seed exudates changed the electrophoretic profiles and content of lipopolysaccharide (LPS) of *R. anhuiense* (Xiang et al., 2020; Wang et al., 2021). Extracts from maize and soybean have been reported to influence the production of lipochitooligosaccharides (Nod factor) in *Bradyrhizobium* (Lian et al., 2002). Previously, we reported that *Bradyrhizobium* strains display different attachment-specific surface properties when grown in soil-extracted solubilized organic matter (SESOM) from soybean fields (Liebeke et al., 2009; Sandhu et al., 2021). SESOM contains a plethora of water-diffusible compounds in soil, the product of degradation compounds, plant exudates, and compound uptake by the microbiota (Vilain et al., 2006; Liebeke et al., 2009). *Bradyrhizobium* has also been reported to express phenotypic variations in flagellar expressions and exopolysaccharide (EPS) composition when cultured with different carbon sources in defined media (Lodeiro et al., 2000; Covelli et al., 2013). All these studies reported the cumulative effects of cocktails of root exudates. However, the effect of individual root exudate compounds (RECCs) from soybean on *Bradyrhizobium* surface properties is minimally explored.

Plants produce a diversity of low-molecular weight compounds consisting of amino acids, sugars, organic acids, phenolics, and other specialized metabolites (Tsuno et al., 2018). Some soybean root exudates function as signaling molecules, while others serve as nutrition for bacteria. Signaling compounds include flavonoids, isoflavones, and soybean saponins, and their role in modifying rhizobial behavior to promote a rhizobia–legume symbiotic relation is well-explored (Sugiyama et al., 2016; Tsuno et al., 2018; Fujimatsu et al., 2020). Further exudates include various carbohydrates, organic acids, and amino acids, but the function of most of these has not been explored (Sasse et al., 2018; Sugiyama, 2019). Root exudate identification of low-K-tolerant and low-K-sensitive cultivars of soybean revealed 43 and 39 metabolites, respectively (Shinano et al., 2020). A study of soybean under phosphorous deficiency revealed 79 compounds in root exudates (Tawaraya et al., 2014). Predominant metabolites included organic and amino acids, specifically gluconate, glutarate, malate, lactate, gentisate, glutamate, aspartate, glycine, and L-serine. Additionally, arabinose and raffinose are abundantly present sugars from soybean roots (Timotiwu and Sakurai, 2002; Shinano et al., 2020). These metabolites can act as a potential carbon source for rhizospheric bacteria and impact their phenotypic properties. In contrast to soybean root exudates as a carbon source in the rhizosphere, *Bradyrhizobium* under laboratory research has been grown on either arabinose, mannitol, or gluconate as a main carbon source (Mathis et al., 1986; Hauser et al., 2007; Cogo et al., 2018). Many comparative studies have reported phenotypic and metabolic changes such as lectin binding, oxygen consumption, exopolysaccharides (EPS), and capsular polysaccharide structure associated with growth in arabinose vs. mannitol (Lodeiro et al., 2000; Hauser et al., 2007; Cogo et al., 2018). Growth on other carbon sources may, therefore, also lead to additional phenotypic differences in *Bradyrhizobium*, for example, root attachment-associated phenotypes. Understanding the effect of individual RECCs of soybean on attachment and attachment-related properties of its symbiotic partner *Bradyrhizobium* could provide a basis for understanding binding to the root.

We hypothesized that individual soybean root exudate chemical compounds impact root attachment-promoting phenotypic properties of USDA 110. We studied (i) the effect of soybean RECCs on the attachment-associated phenotypic properties of USDA 110, an agriculturally important strain of *Bradyrhizobium* in the presence of 11 majorly reported soybean root exudates (Tawaraya et al., 2014; Shinano et al., 2020) and (ii) the relation of different surface and phenotypic properties to the attachment of USDA 110 to soybean roots. We found that various RECCs resulted in substantial surface property differences of USDA 110, including soybean root attachment.

## 2. Materials and methods

### 2.1. Bacterial strains and culture media

*B. diazoefficiens* USDA 110 and *B. japonicum* USDA 20 were obtained from the NRRL Culture Collection of the Agricultural Research Service, United State Department of Agriculture. USDA 110 was tagged with GFP according to Ledermann (Ledermann et al., 2015). Plasmid pRJPaph-gfp, kindly provided by Hans-Martin Fischer, was transformed into *E. coli* S17-1 λ*pir*, and then transferred to USDA 110 using a biparental conjugation peptone-salts-yeast extract (PSY) base. PSY was prepared as described by Mesa et al. (Mesa et al., 2008), and supplemented with an individual carbon source (1 g/L). PSY base was supplemented with one of the 12 different carbon sources (gluconic acid, glutaric acid, malic acid, lactic acid, gentisic acid, aspartic acid, glutamic acid, raffinose, arabinose, mannitol, serine, and glycine) at a concentration of 1 g/L. All different RECC-containing media were set at pH 7.0, and hence names were simplified to gluconate, glutarate, malate, lactate, gentisate, aspartate, glutamate, raffinose, arabinose, mannitol, serine, and glycine. PSY base without peptone and yeast extract was used as a minimal medium, and supplemented with 1 g/L of ammonium chloride and 1 g/L of arabinose. Cultures were incubated at 28°C, and liquid cultures were shaken at 250 rpm. To mimic bulk soil nutritional conditions, we prepared a filter-sterilized soil extract, SESOM (Vilain et al., 2006). Briefly, soybean field soil was dried at 55°C and stored at 4°C. Dry soil (200 g) was added to 1 L of pre-warmed (70°C) sterile MOPS buffer (10 mM, pH 7.0) and shaken for 4 h at 150 rpm. Suspensions were filtered sequentially by using filter paper and cellulose acetate filters of decreasing pore sizes to obtain a clear and sterile extract. The sterile SESOM was supplemented with 0.01 g/L of Bacto-peptone autoclaved separately. For all the following experiments, a 48-h exponential phase culture in an individual carbon source was used as an inoculum.

### 2.2. Yield and colony morphology

To evaluate the growth and yield of USDA 110, it was inoculated to an absorbance of 0.010 (600 nm) into PSY base supplemented with 12 carbon sources separately and in SESOM and incubated at 28°C and 250 rpm for 48 h. After 48 h, 100 μl of the culture was used to perform serial dilutions to 10^−7^, and 20 μl of the 10^−4^-10^−7^ dilutions were inoculated in triplicate on R2A plates according to the droplet plate count method (Lindsay and Von Holy, 1999). Colony forming units were counted after 4 days, and cfu/ml was calculated respective to the dilution.

For colony morphology, 1.5% solid agar plates of PSY base with respective carbon source and SESOM were used. For plate inoculation, USDA 110 was pre-grown in the respective carbon source, diluted to the desired factor, spot inoculated in triplicate on respective media, and incubated at 28°C for 10 days. Images were taken at a standardized distance using an iPhone 10 without any magnification or filters and were cropped to the same size.

### 2.3. Motility and chemotaxis

The effect of different carbon sources on swimming and swarming was determined by spot inoculating 20 μl of USDA 110 pre-grown in the respective carbon source and diluted to an absorbance (600 nm) of 0.100 onto low percentage agar plates. PSY base and the respective carbon source, or SESOM, were supplemented with 0.35% agar for swimming, and with 0.7% agar or swarming. The colony radius was measured after incubating plates for 10 days at 28°C.

The chemotactic response of USDA 110 to all the carbon sources was determined using a capillary tube assay using GFP-tagged USDA 110. GFP-tagged USDA 110 was cultured for 48 h at 28°C in P/10, 10-fold diluted PSY base with peptone (0.3 g/L), and yeast extract (0.1 g/L), but without any carbon source. For chemotaxis, sterile capillary tubes (inner diameter of 1.1–1.2 mm, Fisher Scientific, 22-260943) were filled with a P/10 base supplemented with desired chemoattractant (1 g/L) by placing one end of the tube at an angle of 30° in a Petri plate containing the respective liquid. The other end was plugged by pressing into 3% water agar to prevent liquid from escaping the tube. The open end of the capillary tubes was inserted into a well of a 12-well plate containing cell suspension at an absorbance (600 nm) of 0.500 in the attractant-free P/10 base as described above. The tubes were left dipped in cell suspension at an angle of 45°, ensuring that the open end was submerged in cell suspension but not blocked by the bottom of the well. After 60 min of incubation, capillaries were transferred to a microcentrifuge tube, the agar plug was melted using a Bunsen burner, and the liquid was expelled using a 100 μl tip (Ditty and Parales, 2015). The expelled liquid was transferred to a 96-well-plate, and fluorescence was quantified using a microplate reader (FLUOstar Omega, BMG LABTECH) at excitation and emission maxima of 497/509. Each determination was performed using three technical replicates and repeated on three separate occasions. To increase the sample volume, each technical replicate comprised three capillary tubes. Fluorescence readings were blanked using an uninoculated P/10 base with respective RECC. To quantify chemotactic response, fluorescence accumulation in P/10 without RECC was determined and subtracted from values in the presence of RECC.

Both scanning and transmission electron microscopies were performed to observe the location of expressed flagella. Bacterial samples after 48 h of incubation were harvested gently at 1,000 × g for 20 min. Samples were washed two times without pipetting, and cells were allowed to disperse by gentle swirling of the tube. Samples were resuspended in 5 ml of EMC primary fixative (100 mM sodium cacodylate, 2% glutaraldehyde, and 2% paraformaldehyde) in a 5 ml microcentrifuge tube, incubated at room temperature for 30 min and stored at 4°C. Fixed samples were shipped with an ice pack to ensure that they stayed cool during shipping and were processed at the Electron Microscopy Core Facility at the University of Missouri.

### 2.4. Surface adherence and biofilm formation

Bacterial cell surface hydrophobicity (BCSH) was determined using the MATH assay as previously described (Sandhu et al., 2021). Briefly, USDA110 and USDA 20 cultured in respective media, as described above, were washed with and resuspended in sterile phosphate urea magnesium (PUM) sulfate buffer with pH adjusted to 7.1. Cell suspension (4 mL) was exposed to 1 ml of n-hexadecane (ACROS Organics) by vortexing, and phases were allowed to separate for 1 h. GFP-tagged populations from both aqueous and hydrocarbon phases were investigated by fluorescence microscopy and found to be intact, indicating that n-hexadecane exposure did not cause cell lysis. The absorbance of the n-hexadecane-treated aqueous phase (Absorbance Math: A_M_) and untreated aqueous phase (Absorbance original: A_O_) were measured at 600 nm, and the fraction of cells partitioned to the hydrocarbon phase (F_p_) was calculated as follows: Fp = 1 – (A_M_/A_O_).

The attachment of USDA 110 and USDA 20 to young soybean roots was determined as mentioned previously (Sandhu et al., 2021). Briefly, bacterial populations pre-cultured in specific RECCs were exposed to 9-day-old sterile, root hair expressing roots of length ~8–12 cm for 1 h at room temperature. Before exposing bacterial cells to the root, the culturable count was determined by diluting cell suspensions and using the droplet plate count technique. For counting the attached cells, cell suspension exposed roots were washed three times in sterile water, transferred to conical tubes containing 50 ml of PBS with 0.02% Tween 20, and tubes were exposed to sonication as four 30 s pulses with intermittent cooling in ice for 30 s. Culturable counts before and after sonication showed no significant difference, confirming no impact of sonication on the viability of the cells (data not shown). Cells in suspension were quantified by the droplet plate count method. The ratio of attached cells was calculated as follows: Attached cells/Total cells. To quantify the root attachment of EPS-depleted cells, EPS was removed from mucoid and SESOM-grown populations by 1.5 M NaCl treatment as described in Chiba et al. (2015). For determining the USDA 110 cells attaching to cellulose, the same methodology as above was used, replacing roots with 5 ^*^ 1 cm pure cellulose strips (Whatman Grade 1 filter paper, Cat 1001 125). The total number of cells and attached cells were determined by the droplet plate count method before and after exposing cells to cellulose strips.

Biofilm formation of USDA 110 and USDA 20 grown in different carbon sources and SESOM was quantified on hydrophilic polystyrene as described previously (O'Toole and Kolter, 1998; O'Toole, 2011). Pre-grown 48-h culture in specific media was harvested, resuspended in specific media to an absorbance (600 nm) of 0.100, and 150 μl of cell suspension was loaded in hydrophilic 96-well polystyrene plates (Nunclon Delta Surface Polystyrene). Biofilm was quantified after 48 h of incubation at 28°C, using the crystal violet method as described previously, where the non-inoculated PSY base was used as a blank (O'Toole, 2011).

### 2.5. Lectin binding assay

Surface-exposed sugars were characterized using 12 lectins: wheat-germ agglutinin (WGA), *Vicia villosa* agglutinin (VVA), *Ulex europaeus* agglutinin I (UEA I), succinylated wheat-germ agglutinin (SWGA), *Solanum tuberosum* lectin (STL), soybean agglutinin (SBA), *Ricinus communis* agglutinin I (RCA I), peanut agglutinin (PNA), *Griffonia simplicifolia* lectin I (GSL I), *Galanthus nivalis* Lectin (GNL), *Datura stramonium* Lectin (DSL), and Concanavalin A (ConA) (Vector Laboratories, Burlingame, CA 94010, USA). Some of the above-mentioned lectins were fluorescein-conjugated, and some were biotinylated. Lectin binding assay was performed as previously mentioned (Sandhu et al., 2021). Briefly, 48-h old culture in the PSY base with different carbon sources and SESOM were harvested and washed with HSB buffer (2.383 g/L of HEPES, 8.766 g/L of NaCl, 0.011 g/L of CaCl_2_, and 0.8 g/L of sodium azide) and resuspended to an absorbance (600 nm) of 0.100. In total, 50 μl of prepared cell suspension was added to 100 μl of lectin at a concentration of 20 μg/ml in HSB, vortexed, and incubated for 20 min. Cells were harvested and washed three times with HSB. Fluorescein-conjugated lectin-processed cells were resuspended and transferred to a microscopic slide. Biotinylated lectin-processed cells were supplemented with 50 μl of 20 μg/ml of streptavidin-FITC and incubated for another 30 min and later processed as mentioned for others. An Olympus BX53 Upright Compound Microscope was used to observe prepared slides at 446–486 nm excitation and a 498–552 nm emission filter, and images were captured using an Olympus DP70 digital camera. The percentage of cells binding to lectins was determined by counting cells using Image J.

### 2.6. Exopolysaccharide quantification

USDA 110 grown in the PSY base supplemented with a specific carbon source and SESOM was grown for 48 h at 28°C. Trichloroacetic acid (TCA) was added to the cell suspension to a final concentration of 10%, and the mixture was stirred for 45 min at 22°C (Miao et al., 2014). Cells and TCA-precipitated proteins were pelleted at 10,000 rpm for 45 min at 4°C. Supernatant was mixed with 100% ethanol at a 1:3 ratio, and the mixture was incubated overnight at 4°C. Precipitated EPS was pelleted by centrifugation for 45 min at 4°C, and pellets were washed two times with 83% ethanol, dried by vacuum centrifugation, and stored at −20°C. EPS was quantified as described (Morris, 1948; Xu et al., 2021). Briefly, dried EPS pellets were suspended in 1 ml of sterile water, and three aliquots of 30 μl were mixed with 83 μl of anthrone reagent (0.01 g anthrone, 0.5 ml of ethyl acetate, and 5 ml of concentrated H_2_SO_4_). Aliquots were incubated at 100°C for 4 min and incubated on ice for 10 min. Samples were loaded into a 96-well plate, and the absorbance was measured at 620 nm with a microplate reader. For the negative control, 30 μl of sterile water was used. For quantifying the EPS, absorbance from negative control was subtracted from test samples, and a known glucose standard served as a reference (Morris, 1948).

### 2.7. Lipopolysaccharide profiling

For lipopolysaccharide, USDA 110 was grown in PSY base supplemented with a specific carbon source and in SESOM for 48 h, and cells were harvested by centrifugation at 7,000 rpm for 20 min, washed, resuspended in sterile water, and harvested at 7,000 rpm for 10 min. Isolation of LPS was performed as described by Davis and Goldberg (2012). LPS was obtained in 200 μl of 2x SDS. LPS was resolved by loading 65 μl into 16% SDS PAGE gel. LPS was visualized by silver staining as described by Tsai and Frasch (1982), as modified by Preston and Penner (1987).

### 2.8. Statistical analysis

All the experiments in this study were performed in a set of three biological replicates, where each biological replication comprised three technical replicates, except biofilm formation which had seven technical replicates. To test the significant difference in the observation of different carbon sources, Fisher's least significant difference (LSD) test was performed in R (R Core Team, 2021). Pearson's correlation was estimated in R (R Core Team, 2021) to analyze the relationships between the surface properties to the attachment-related properties in the presence of compounds of soybean root exudates. We used several packages in R, including *dplyr, agricolae, ggplot2, ggpubr*, and *corrplot*, for exploratory data analysis and visualization.

## 3. Results

*B. diazoefficiens* USDA 110 displayed unique surface and attachment-related phenotypes when grown in different compounds found in soybean root exudates. This spanned motility, flagellar location, chemotaxis, mucoidy and quantity of EPS, LPS profile, BCSH, biofilm formation, attachment to cellulose as a major young root component, and attachment to the soybean root. Some RECCs promoted root attachment. Our results indicate that USDA 110 displays a high degree of RECC-specific root attachment-related phenotypic plasticity in its surface properties. Each RECC impacts different attachment-related properties specifically. Another good nitrogen fixer, *B. japonicum* USDA 20, displayed similar cell surface hydrophobicity, biofilm formation, and root attachment properties in various RECCs.

### 3.1. Exudate compounds support the growth of USDA 110

**Figure 2:**
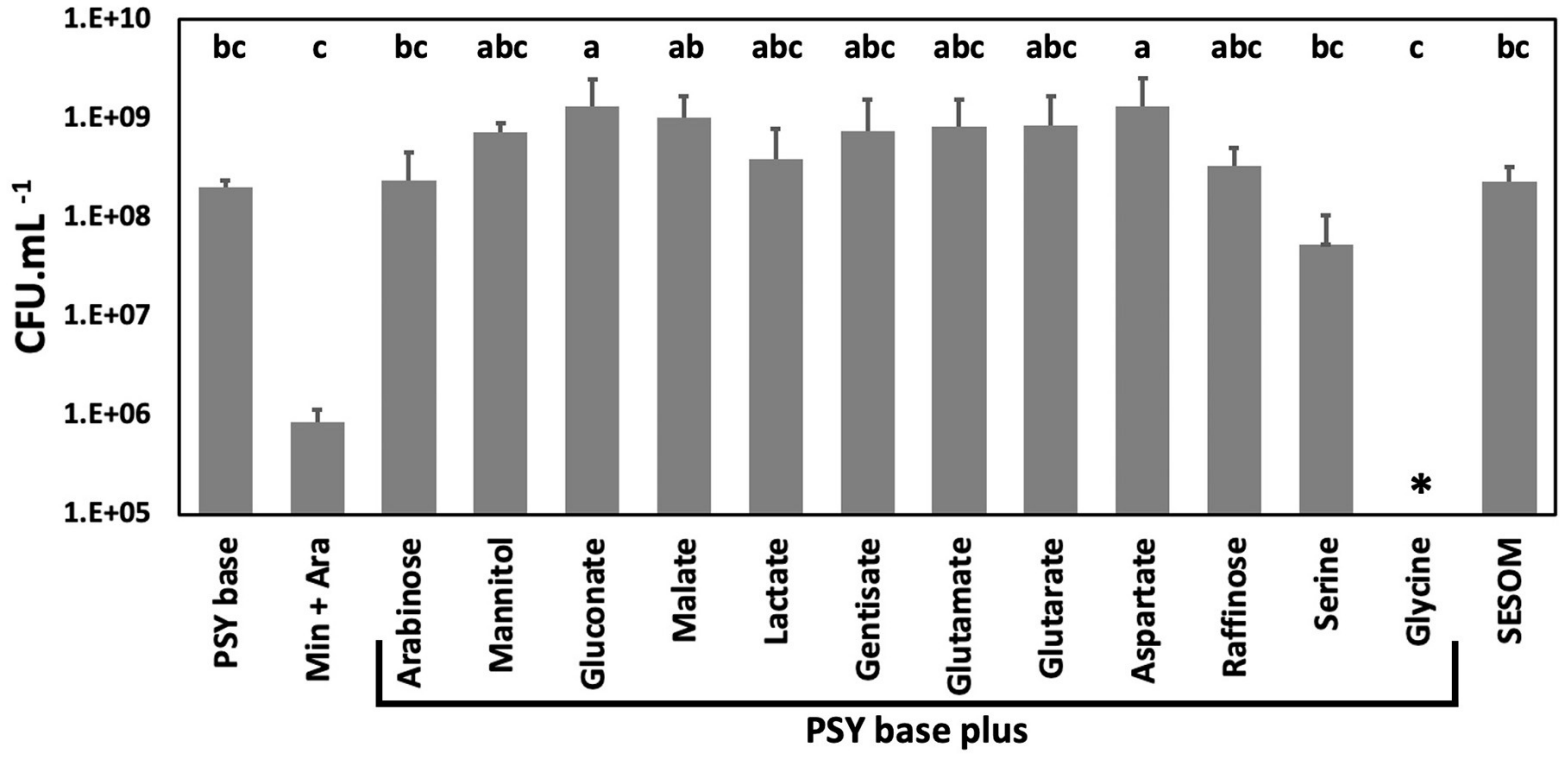
Biomass yield of *B. diazoefficiens* USDA 110 grown to early stationary phase in liquid media while shaking at 28°C. Yield was determined in PSY (Peptone salt yeast extract) base without and with 12 carbons sources, in PSY with arabinose but without yeast extract and peptone (Min + Ara), and in soybean field soil-extracted soluble organic matter (SESOM). Error bars indicate one standard error of the mean, and superscripts indicate grouping as determined by ANOVA. *No biomass increase could be detected with glycine as a carbon source.

To determine biomass yield, USDA110 was inoculated into PSY base and PSY base without peptone and yeast extract supplemented with ammonium chloride (Min + Ara). PSY base without peptone and yeast extract and with arabinose as a carbon source supported limited growth as compared to the PSY base alone (Figure 2). Therefore, PSY base instead of the minimal medium was used for phenotype analysis to confirm that the observed phenotype is because of the effect of the root exudate and not because of growth limitations. For this study, the PSY base was supplemented with 11 RECCs individually, reported to be predominant in soybean root exudate (Timotiwu and Sakurai, 2002; Tawaraya et al., 2014; Shinano et al., 2020), and mannitol, a widely used carbon source for *Bradyrhizobium*. Bulk soil nutritional conditions were mimicked using a filter-sterilized soybean soil extract, SESOM. Biomass yield was similar across all carbon sources except for a lower yield on serine, while glycine did not support growth (Figure 2). The biomass yield was lower in PSY-glycine than PSY alone, suggesting that glycine inhibits the growth of USDA 110.

### 3.2. Root exudates affect motility and chemotactic response

The effect of root exudates on motility-related properties was quantified because chemotaxis and motility play roles in successfully attracting bacteria to the root. Phenotypic plasticity in swimming and swarming ability and flagellar expression was observed when USDA 110 was grown in different RECCs. Each of the compounds also led to a different degree of chemotaxis.

**Figure 3:**
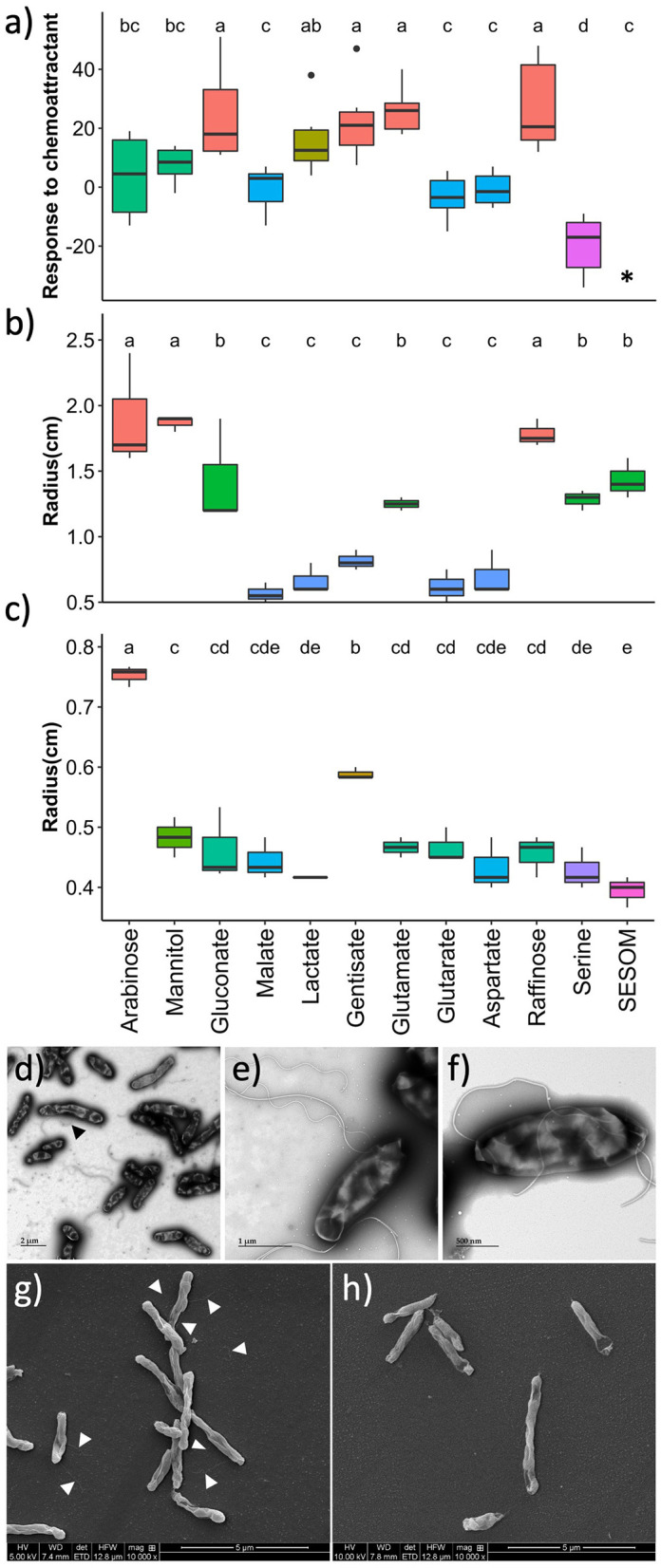
Motility of *B. diazoefficiens* USDA 110 growing in soybean root exudate compounds, measured by **(a)** chemotaxis, **(b)** swimming, and **(c)** swarming. Transmission electron microscopy of cells grown in arabinose with the black arrow pointing to a lateral flagellum **(d, e)**, and of cells grown in gentisate **(f)**. Scanning electron microscopy of cells grown in arabinose with white arrows pointing to flagella **(g)**, and of cells grown in soil-extracted soluble organic matter (SESOM) **(h)**. *Chemotaxis could not be measured in SESOM due to the autofluorescence of the medium.

Chemoattraction was quantified by placing capillary tubes containing RECCs into suspensions of GFP-tagged USDA 110 cultured in PSY without exudates. Accumulation of fluorescent cells in the capillary indicated chemotaxis toward the compound. Gluconate, gentisate, glutamate, and raffinose displayed higher chemoattractant ability, while arabinose, mannitol, and lactate were weaker attractants (Figure 3a). Malate, glutarate, and aspartate did not lead to attraction, while serine acted as a repellent. The attractant ability of SESOM could not be measured because of the high broad-spectrum autofluorescence of the medium itself.

To determine the influence of exudate compounds on the swimming and swarming of USDA 110, populations were spot inoculated onto 0.35 and 0.7% agar plates, respectively. Both the pre-culture medium and assay plate had been supplemented with the same RECC. Arabinose, mannitol, and raffinose supported the highest degree of swimming, followed by glutamate, glutarate, serine, and SESOM (Figure 3b). The remaining five root exudates supported swimming poorly. Only arabinose, and to a lesser degree, gentisate promoted swarming. None of the other compounds supported swarming beyond the zone of inoculation (Figure 3c). The different polarities of the RECC agars influenced the spreading radius of the 20 μl of inoculum on the agar surface. For example, SESOM spread out the least, leading to the smallest radius at t_0._ Due to variations observed in the motility-related properties, we decided to determine the flagella type and location by electron microscopy.

Swimming in USDA 110 requires subpolar flagella while swarming requires lateral flagella (Covelli et al., 2013). To corroborate swimming and swarming results, we determined the flagellar location and type in selected exudates: arabinose for high swimming and swarming, raffinose for high swimming and lack of swarming, gentisate for swarming and lack of swimming, and SESOM for limited swimming and lack of swarming. Arabinose-grown cells expressed one form of lateral and two forms of subpolar flagella (thin and thick, Figures 3d, e). Gentisate and raffinose displayed thick subpolar flagella but no thin subpolar or lateral flagellum (Figure 3f; Supplementary Figure S1). For SESOM, a very small proportion of the cells expressed subpolar flagella only as compared to many in arabinose (Figures 3g, h). These results indicated that swimming, swarming, chemotaxis, and flagellum type are each influenced individually by each specific RECC. Arabinose, gentisate, and raffinose were the RECCs with the strongest impact on motility and chemotaxis traits, while malate, glutarate, and aspartate lead to immobile cells. Thus, arabinose and gentisate both promote swimming and swarming, and gentisate and raffinose both are good chemoattractants.

### 3.3. Attachment traits vary with root exudates and the nature of the attachment surface

**Figure 4:**
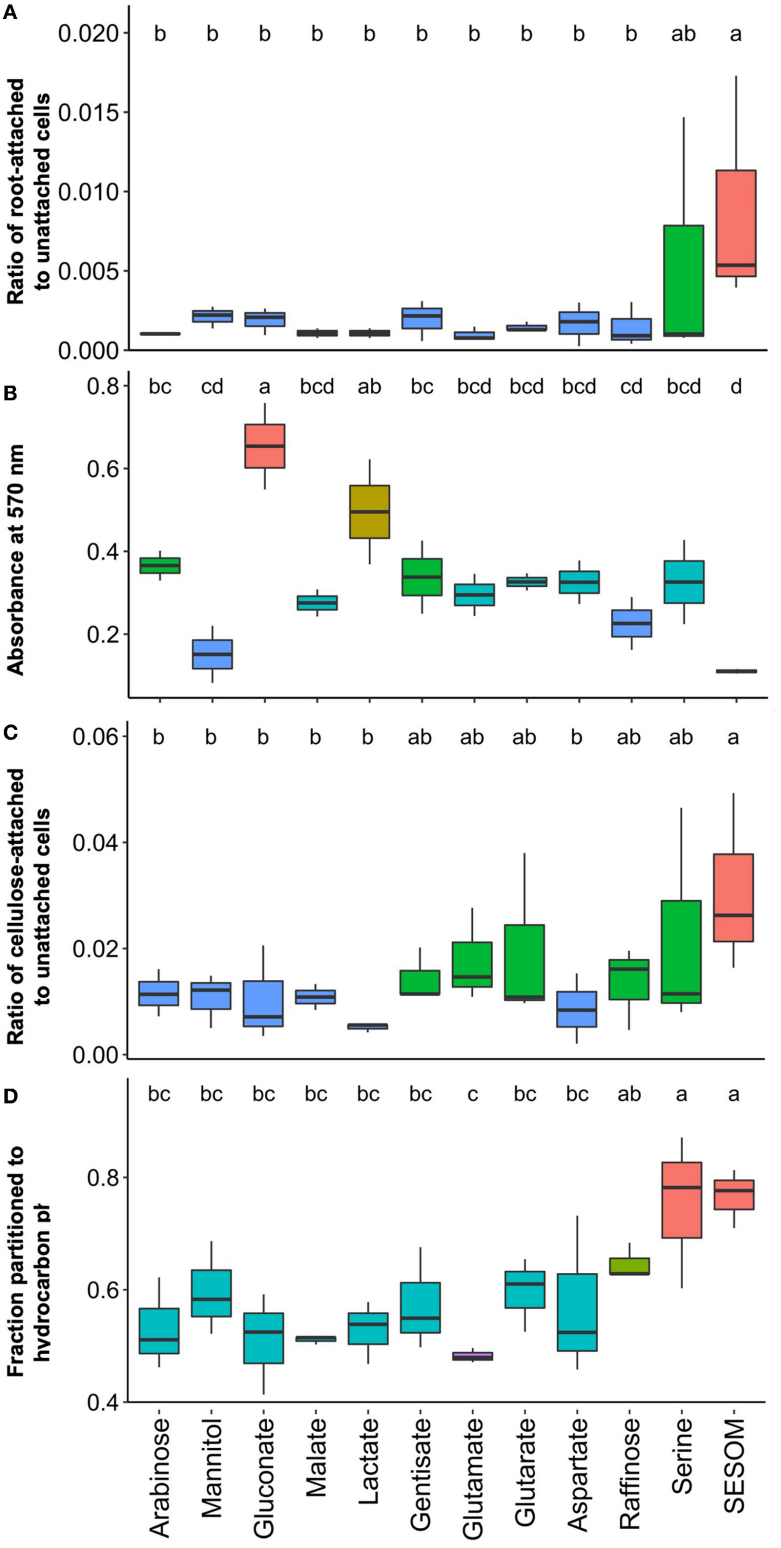
Attachment properties of *B. diazoefficiens* USDA 110 measured by **(A)** attachment to soybean roots, **(B)** biofilm formation on hydrophilic polystyrene, **(C)** attachment to cellulose, and **(D)** surface hydrophobicity determined by the MATH assay.

Attachment and attachment-related properties of USDA 110 were affected significantly by the different RECCs. To imitate natural conditions, we measured attachment to soybean root and cellulose, one of the main young root components. We compared this to standard biofilm formation on hydrophilic polystyrene and to surface hydrophobicity. To determine whether other good nitrogen fixers behave similarly, we also tested biofilm formation, cell surface hydrophobicity, and soybean root attachment for USDA 20 populations grown in arabinose, mannitol, serine, and SESOM. Root attachment was determined by dipping 9-day-old gnotobiotic seedlings in exponential phase cultures for 60 min, and quantifying cells were dislodged by sonication. Serine and SESOM-grown populations had significantly higher soybean root-binding ability than all the other exudate-grown populations (Figure 4A). While poorly adhering populations displayed low and consistent proportions of attaching cells, serine and SESOM attachment numbers varied widely. This suggests heterogeneity in the proportion of adherence-able cells in serine and SESOM populations. USDA 20 also displayed a low root-binding percentage when grown in arabinose and mannitol, and a high root-binding percentage of cells when grown in SESOM. Unlike USDA 110, USDA 20 serine-grown populations displayed limited root binding (Supplementary Figure S2). Serine only promoted root attachment for USDA 110 and not 20.

**Figure 5:**
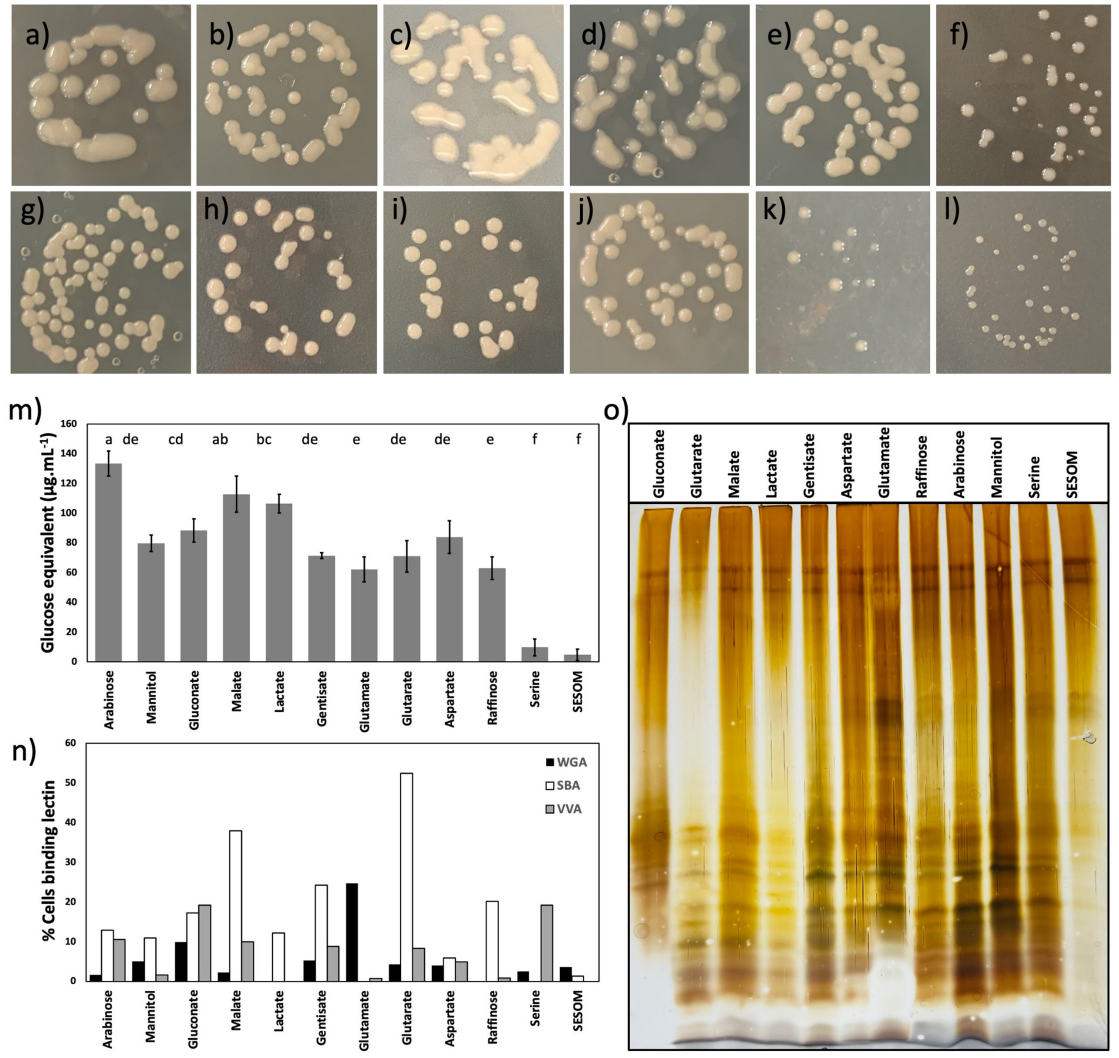
Colony morphology of *B. diazoefficiens* USDA110 on PSY agar supplemented with arabinose **(a)**, mannitol **(b)**, gluconate **(c)**, malate **(d)**, lactate **(e)**, gentisate **(f)**, glutamate **(g)**, glutarate **(h)**, aspartate **(i)**, raffinose **(j)**, serine **(k)**, and soil-extracted soluble organic matter (SESOM) **(l)**. Surface saccharides of *B. diazoefficiens* USDA 110 grown in liquid culture **(m)**, and binding of soybean agglutinin, wheat-germ agglutinin, and VVA to exponential phase liquid cultures **(n)**. Lipopolysaccharide profiles of populations grown in the liquid culture **(o)**.

To quantify biofilm formation, we used hydrophilic polystyrene 96-well plates, and the formed biofilm was quantified after 48 h by crystal violet staining. The highest biofilm-forming ability was observed in arabinose, gluconate, lactate, and gentisate-grown populations (Figure 4B). The first three of these biofilm-promoting compounds were also observed to support high colony mucoidy (Figures 5a, c, e). No biofilm was detected in SESOM, and very little was formed in mannitol or raffinose. Longer incubation in SESOM did not result in biofilm formation (data not shown). Surprisingly USDA 20 did not form biofilm on any of the tested root exudates and SESOM (Supplementary Figure S2a only shows four compounds, remaining data not shown). The attachment of USDA 110 to cellulose was determined using filter paper rectangles, and the data mirrored root attachment results (Figures 4A, C). SESOM and serine displayed the highest cellulose attachment ability, similar to root attachment (Figure 4C). Raffinose-, gentisate-, glutamate-, and glutarate-grown populations attached more to cellulose than to roots.

Cell surface hydrophobicity, measured as partition into hydrocarbon, followed the same general pattern as for root and cellulose attachment. SESOM and serine both resulted in populations with high cell surface hydrophobicity followed by raffinose. The other populations had significantly similar and moderate levels of surface hydrophobicity, and the lowest was glutamate (Figure 4D). USDA 20 SESOM-grown populations were also highly hydrophobic in nature, but unlike USDA 110, serine-grown populations were not (Supplementary Figure S2b). The root and cellulose attachment results indicated that serine and SESOM-grown cells of USDA 110 are more hydrophobic in nature, and the hydrophobicity assay data confirmed this. A similar observation was made with USDA 20, where SESOM-grown cells were highly hydrophobic and had the highest proportion of root-binding cells. These results indicate a direct relation for cell surface hydrophobicity to root and root component attachment. However, biofilm formation was highest for hydrophilic populations and contrasted with root attachment for both USDA 110 and 20. This is likely due to the difference in nature and hydrophilicity of root and polystyrene surfaces. However, even the same hydrophobic nature of both root and polystyrene resulted in different anchoring ratios, probably because of other differences in surface (Bashan and Holguin, 1993).

### 3.4. Exopolysaccharide and lipopolysaccharide vary with root exudate compound

We first characterized the colony morphology of USDA 110 by plating dilute, exponential phase cell suspension on PSY agar with individual RECCs, and SESOM. The color of the culture medium was affected by individual RECCs (Figures 5a–l). USDA 110 displayed remarkable variation in the colony size and mucidness when grown on the various individual RECC. SESOM and serine supported smaller and less mucid colonies, whereas malate, lactate, gluconate, and arabinose supported large and highly mucoid colonies (Figures 5a–l). As the cell yield in the liquid medium did not differ significantly for any growth-supporting compound but serine (Figure 2), the larger colony sizes were more likely due to extracellular materials than to growth yield. The visual appearance of highly mucoid colonies varied according to the RECCs, suggesting differences in EPS structure and composition.

To corroborate apparent differences in exopolysaccharide production in different RECCs, we quantified extractable EPS and characterized surface-exposed sugars by semi-quantitative lectin profiling. We found significant differences in EPS quantity produced, and this supported visual observations of colony mucoidy (Figure 5m). Arabinose, gluconate, malate, and lactate supported the highest quantity of EPS, and the most mucoid colonies (Figures 5a, c–e, m). Mannitol, gentisate, glutamate, glutarate, aspartate, and raffinose supported comparatively less EPS, while SESOM and serine produced almost none, mirroring colony morphology (Figures 5k–m). The EPS-depleted cells (NaCl-treated cells) had no significant difference in root attachment when compared to non–NaCl-treated cells (Supplementary Figure S3).

Of the 12 lectins applied, only soybean agglutinin (SBA) lectin, wheat-germ agglutinin (WGA) lectin, and *Vicia villosa agglutinin* (VVA) lectin bound to almost all different RECC grown USDA 110 populations. Binding of the other nine lectins occurred for one or more RECC, but only to < 1% of the population (data not shown). Lectin binding varied according to the RECC used (Figure 5n). Surprisingly, serine- and SESOM-grown populations displayed no binding to SBA; the soybean agglutinin lectin was believed to play a role in the initial attachment of *B. diazoefficiens* to the soybean root surface. In contrast, glutarate, malate, arabinose, mannitol, gentisate, and other RECCs led to SBA binding, and glutarate- and malate-grown populations had the highest SBA binding ability of ~40–50%. Similarly, VVA and WGA did not bind all population types, indicating structural differences in EPS due to growth using different RECCs. We were surprised to observe that lectin binding was not universal but only occurred in a fraction of the population. Application of 10 times the lectin concentration did not alleviate the occurrence of un-bound cells, indicating that this was not due to insufficient lectin. The clonality of populations was confirmed regularly through plate culture and microscopy. The apparent heterogeneity of lectin binding to single-strain exponential phase populations suggests phenotypic heterogeneity and this should be further explored.

Surface-exposed sugars can be part of EPS or lipopolysaccharides (LPS), so we determined the electrophoretic LPS profiles of the 12 populations (Figure 5o). Only minor differences in the LPS profile were observed. All 12 profiles, except SESOM, showed a range of bands usually assigned as O-antigen chains of different lengths. LPS extracts were obtained from cell suspensions normalized for density by optical density, and the upper two bands indicate parity in the loading of extract. These results suggest that SESOM-grown cells have much less O-antigen-containing LPS than the other 11. Taken together, EPS and LPS varied by RECC grown on, pointing to the phenotypic plasticity of *B. diazoefficiens* USDA 110.

### 3.5. Correlation of phenotypes across the root exudate compounds

**Figure 6:**
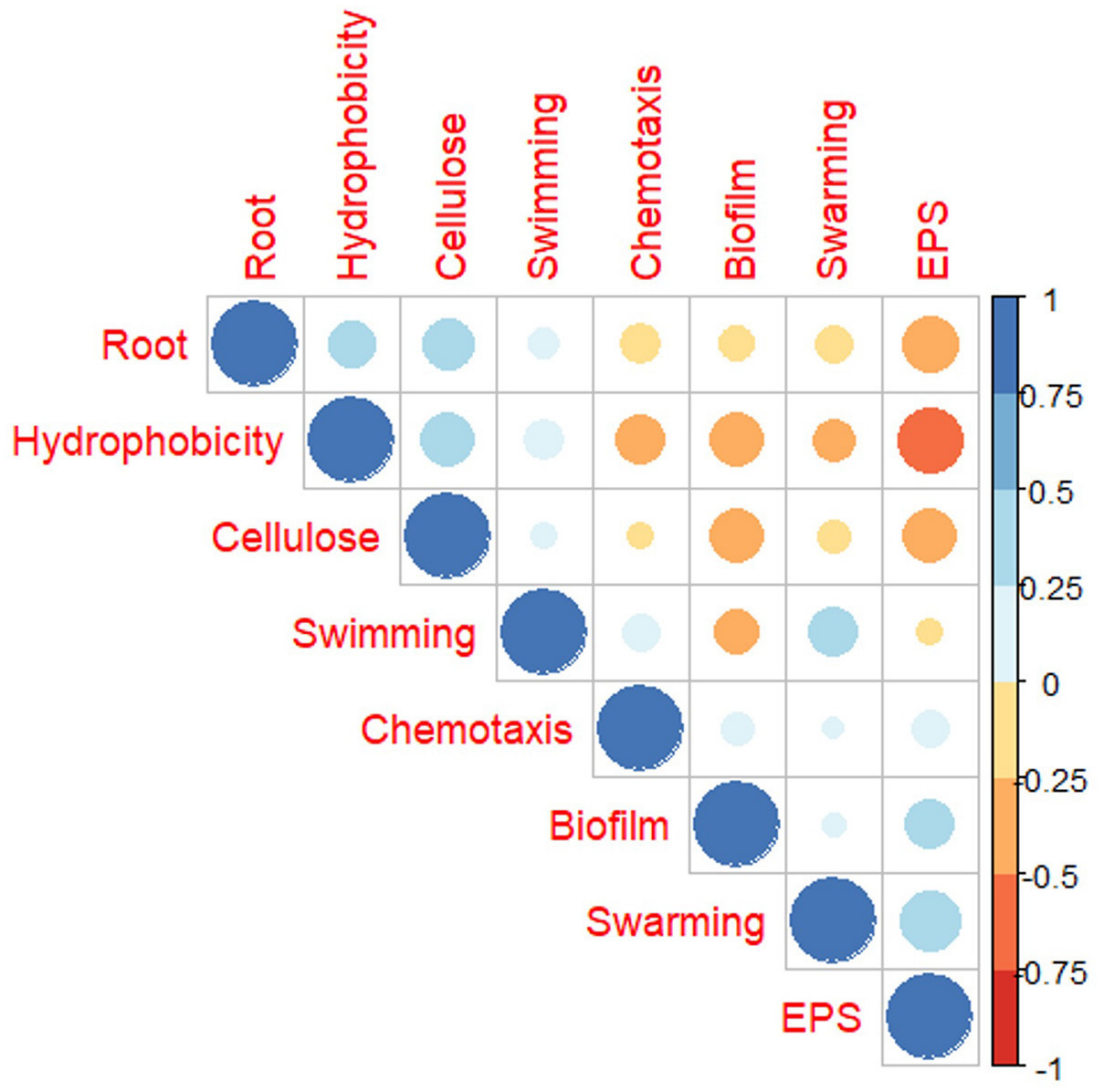
Pearson's correlation of *B. diazoefficiens* USDA 110 surface and attachment properties when grown in different root exudate compounds.

To understand the relation of the root adherence ability of USDA 110 with other surface properties across the 12 RECC, we calculated Pearson's correlation coefficient. Observations from all the adherence and phenotypic properties from all the carbon sources were used to study the correlation. As observed in Figure 6, root adherence ability correlated positively with cellulose attachment and cell surface hydrophobicity of the *Bradyrhizobium*. In contrast, root adherence negatively correlated with the exopolysaccharide quantity and biofilm formation ability. The motility-related properties, including swimming, swarming, and chemotaxis seemed to have minor or no relation to the root attachment ability of USDA 110. Another important observation was that exopolysaccharide and hydrophobicity were highly negatively correlated with one another. Both biofilm formation and EPS quantity are often linked to nodule formation ability but were negatively correlated to almost all the other adherence-related properties except for themselves.

## 4. Discussion

Plant root exudates play an important role in cellular pathways and gene expression of rhizosphere bacteria (Mahmoud and Angle, 1987; Liu et al., 2017; Mavrodi et al., 2021), but the role of individual members of these exudate cocktails on symbiotic relationship development with *Bradyrhizobium* has not been investigated. Here, we evaluated how 11 major soybean root exudate compounds (RECCs) and SESOM (soil-extracted solubilized organic matter) impacted the attachment of *Bradyrhizobium* to the root. We found that 10 (arabinose, gluconate, glutarate, malate, lactate, gentisate, aspartate, glutamate, raffinose, and serine) of them supported growth along with SESOM. Glycine did not support growth. The 10 growth-supporting root exudates affected the attachment-associated phenotypic properties of *B. diazoefficiens* USDA 110 in unique ways. Adaptable cell surface phenotypes provide cells with surface traits that are used to interact with their environment. For example, cancer cells can mask themselves with sialoglycans which inhibit immune cell activation. Expression of this specific phenotype opened a gateway to cancer treatment by representing a to be targeted “glycoimmune checkpoint” (Gray et al., 2020). The phenotype of the cell surface and its importance remains widely unexplored in agricultural microbiology. In this study, we observed major surface phenotypic changes in USDA 110 in response to specific RECCs, impacting root adhesion. This suggested that USDA 110 is very responsive to its nutritional environment. The most obvious phenotypic difference can be seen in the colony morphology of USDA 110. The relationship between different phenotypic properties to the root attachment of USDA 110 varied significantly according to the RECC. Although cell surface properties required for nodule formation are known to play a role in developing successful rhizobia–legume interaction, their specific function and relation to attachment often remain overlooked (Vicario et al., 2015; Wheatley and Poole, 2018).

The movement of rhizobia toward the root is the first step toward root colonization and likely involves chemoattraction by root exudates or other signals (Tambalo et al., 2015). After confirming growth in 10 of 11 RECCs and SESOM, we analyzed their impact on chemotaxis- and motility-related properties. Root exudates are reported as better natural chemoattractants when compared to seed exudates and isoflavones, which do not enhance chemotaxis (Barbour et al., 1991; Compton and Scharf, 2021). Liu et al. reported increases in motility- and chemotaxis-related proteins of *B. diazoefficiens* when exposed to soybean root exudates (Liu et al., 2015). Gluconate, gentisate, glutamate, and raffinose displayed high chemoattractant ability. Root exudate glutamate has previously also been reported to be a strong chemoattractant for *B. japonicum* USDA 110, 634b, and 10K. Similar to our results, serine was reported to be a negative chemoattractant for USDA 634b and a very weak chemoattractant for USDA 110 (Barbour et al., 1991; Chuiko et al., 2002; Quelas et al., 2016). Raffinose and gentisate were also observed to promote swimming and swarming abilities, respectively, when compared to the laboratory-used carbon sources, arabinose and mannitol. Along with raffinose, gluconate, glutamate, and serine also promoted the swimming of USDA 110, whereas gentisate was the only root exudate that caused swarming. Peanut seed exudates have been reported to increase swimming motility in five rhizobial strains whereas an increase in swarming was observed in just one of the strains (Vicario et al., 2015). Arabinose also promoted swarming and has previously been reported to express swarming-promoting lateral flagella along with two subpolar flagella in *Bradyrhizobium* (Kanbe et al., 2007; Covelli et al., 2013). Based on this, we anticipated gentisate-grown populations to also express lateral flagella, but we failed to find any lateral or thin flagellum on gentisate-grown cells, suggesting some other swarming-promoting phenomenon. Strangely, the SESOM-grown population displayed swimming similar to several other root exudates, but very few cells had flagella on their surface. It is possible that the flagella of SESOM-grown cells were less resilient and were detached during the wash steps for SEM. Based on motility, raffinose and gentisate are colonization-promoting RECC as they are both chemoattractants and motility promoting for USDA 110.

Once at the root, rhizobia attach to and colonize the root surface. Although root attachment is exudate mediated, this area stays overlooked, and we failed to find any soybean root exudates and root attachment-related studies (Swamy et al., 2016). However, growth on root exudates of *Brachypodium distachyon* regulates surface attachment-related genes in *P. synxantha* 2-79 and *P. fluorescens* SBW25 (Mavrodi et al., 2021). We first analyzed whether any of the RECCs promote attachment to the root surface, and followed this by analyzing attachment-related phenotypic properties. All the RECCs except serine had similar low-level binding to the root, but serine and SESOM surprisingly caused a significantly high ratio of root-attaching cells for USDA 110. SESOM-grown USDA 20 also displayed higher root attachment properties but did not when grown in serine. Attachment to cellulose, a predominant surface component of young roots, followed the same pattern as root attachment (Fang, 2006). Gentisate, glutamate, glutarate, and raffinose did support cellulose but not root attachment for USDA 110. These results indicate that serine is a root and cellulose attachment-promoting exudate for USDA 110.

Bacterial cell surface hydrophobicity (BCSH) is touted as one of the major non-specific factors promoting root attachment, as bacteria are believed to bind to hydrophobic molecules on roots (Wheatley and Poole, 2018; Knights et al., 2021). Much has been published on the role of BCSH in abiotic surface attachments, but we found no studies focusing on root surfaces (Czaczyk et al., 2008; Mazumder et al., 2010; Krasowska and Sigler, 2014). Hydrophobic mutants of *B. japonicum* 138NR had a better ability to compete for nodule occupancy (Yagi et al., 2000). Our BCSH analysis of USDA 110 revealed that populations grown in different RECCs had similar levels of BCSH, except for serine. SESOM caused a significantly high level of surface hydrophobicity in both USDA 110 and 20. The RECCs caused similar profiles of root and cellulose attachment and BCSH, and cellulose and root attachment were positively correlated with bacterial cell surface hydrophobicity by Pearson's coefficient. These results indicate that BCSH is one root attachment promoting phenotype of *Bradyrhizobium*.

After primary attachment, bacteria colonize the root surface by establishing biofilm structures. Therefore, we evaluated the biofilm-promoting ability of RECCs and its correlation with root attachment. In contrast to the attachment to roots, cellulose, and hydrocarbon, biofilm formation of SESOM- and serine-grown populations on hydrophilic polystyrene was negligible, even after 5 days of incubation, and for both strains (data not shown). Among the RECCs, gluconate and lactate promoted biofilm formation on polystyrene plates for USDA 110. However, neither of these compounds had any influence on attachment to the root surface. In contrast, SESOM-grown populations had the highest root attachment but did not form biofilms. Surprisingly, USDA 20 formed no biofilm in any of the 12 tested RECC compounds (data shown only for 4). Correlation analysis revealed a negative correlation between root attachment and biofilm formation in 96-well polystyrene plates. Perez-Gimenez et al. (2009) observed that the non–biofilm-forming mutant USDA 110 ΔP22 had impaired root adhesion. Biofilm development follows primary root attachment; hence, we question the use of the polystyrene plate assay to infer the root attachment behavior of *Bradyrhizobium* to soybean roots.

After developing knowledge about the attachment and biofilm formation behavior of USDA 110, we studied attachment-promoting envelope-associated polymers. These polymers include lipopolysaccharides, a major factor contributing to the rhizobium cell surface hydrophobicity, and exopolysaccharides that are major factors contributing to biofilm formation ability (Park and So, 2000; Fraysse et al., 2003; Perez-Gimenez et al., 2009; Bogino et al., 2013). The EPS quantification results were relatable to colony morphology and biofilm formation ability of the *Bradyrhizobium*. As expected from the non-mucoid colony morphology, serine and SESOM-grown populations have extremely low EPS production but surprisingly the highest root attachment. High EPS-producing populations depleted of EPS by sodium chloride washing did not decrease the root attachment ability. This strongly suggests that exopolysaccharide may not play a significant role in the primary attachment of USDA 110 but that another factor is involved. In another study, an EPS-deficient mutant of *Rhizobium* was very successfully able to form biofilm on the roots of non-legume host plants *Arabidopsis thaliana* and *Brassica napus*, indicating that EPS is not crucial in biofilm formation (Santaella et al., 2008). In another study, altered EPS mutants of *B. japonicum* 2143 also had no effect on the nodule formation ability of the host (Karr et al., 2000). Along with the role of EPS, other bacterial biofilm formation-inducing factors have been reported, such as electrostatic forces and flagella (Rinaudi and Giordano, 2010; Deev et al., 2021). Lectin-binding data also showed phenotypic plasticity as each RECC had its own lectin-binding barcode profile. This indicated that RECC-specific populations had unique sugar groups exposed at the surface. Malate and Glutarate grown cells bound SBA, indicating α- or β-linked terminal GaINAc, GalNAc α-1,3 Gal at the cell surface. While soybean roots are reported to present the SBA lectin at their surface, malate- and glutarate-grown cells displayed poor root attachment. This may be because SBA was not present at the surface of our young root system, due to age, growing conditions or variety, or a combination of these. Conversely, serine-grown populations did not bind SBA whereas they displayed high root attachment. A similar observation was made when a change in the composition of exopolysaccharide and reduction in SBA binding was observed by *B. japonicum* 2143 when grown in malate or arabinose and mannitol as a carbon source but the nodule-forming ability was unaltered (Karr et al., 2000). Plant lectins are also known to develop successful attachment of the rhizobia with the plant (Hoff et al., 2009), but our results suggest that soybean root lectins are not obligately required for *Bradyrhizobium* attachment. While each RECC has its own lectin binding profile, only a part of these populations bound to the specific lectin, suggesting that only a proportion of cells expressed the lectin-specific sugar at its surface. This was surprising as it indicates phenotypic heterogeneity in homogenously grown populations. Although VVA and WGA are not related to soybean attachment, variation in their attachment in different carbon sources also indicated phenotypic heterogeneity in USDA 110.

Lipopolysaccharide impacts the nodulation of soybean as LPS mutants nodulate soybean poorly and have increased BCSH, suggesting a direct relation between LPS structure and BCSH (Chang et al., 2008; Noh et al., 2015). Despite observing significant differences in BCSH, we observed only minor differences in the electrophoretic profile of LPS extracts of the various populations. Enhanced hydrophobicity in SESOM and serine could be due to decreased cellular EPS that exposes the hydrophobic LPS core to the environment which otherwise stays hidden because of EPS in other populations. A possible additional factor impacting cell surface hydrophobicity is a Nod factor, a group of lipochitooligosaccharides with a long lipid tail. Nod factors are best known for their role in legume–rhizobium communication (Cullimore et al., 2001). Plant-secreted isoflavones induce the expression of *nodABC* via the activation of NodD, and the resulting Nod factor induces the uptake of *Bradyrhizobium* to form nodules. Nod factor has been obtained from culture supernatant (Ehrhardt et al., 1996), implying that it is secreted from bacteria. Yet the apolar nature of the Nod factor suggests retention at the cells' surface rather than dissolution into the aqueous phase. Only one study has reported on the extraction of Nod factors from the cells, and it reported that most of the Nod factors accumulate primarily in the membranes of *R. leguminosarum* sv trifolii. Any Nod factor in the supernatant was below the detection limit (Orgambide et al., 1995). Recently, NodD2, a naturally active second copy of *nodD* was identified in *R. leguminosarum* bv. trifolii which might enable Nod factor production without flavonoids as it exhibited flavonoid-independent transcriptional activation (FITA) of the *nodA* gene (Ferguson et al., 2020). Whether the Nod factor contributes to BCSH and initial root attachment should be addressed in future studies.

**Figure 7:**
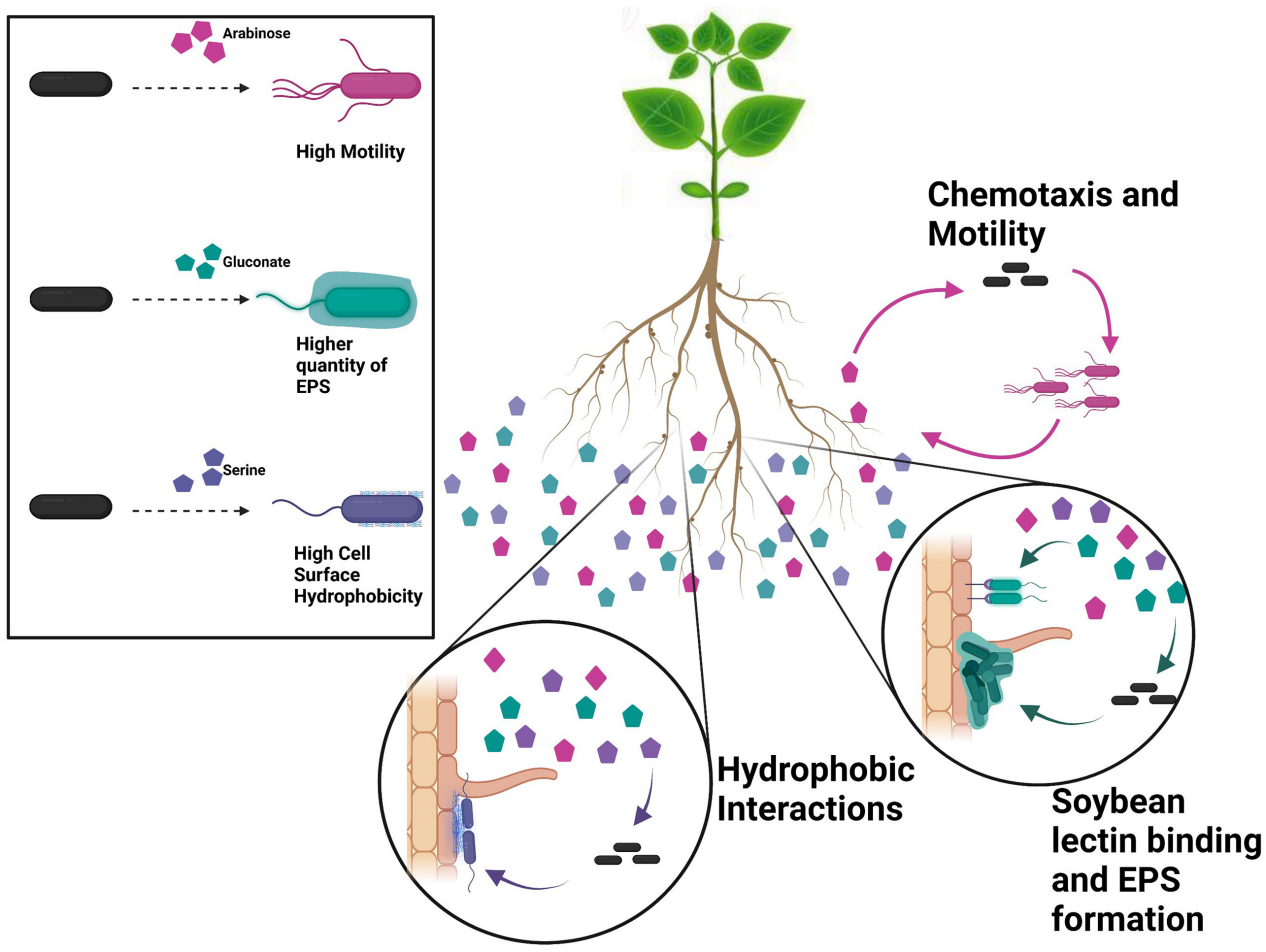
Our data indicate that USDA 110 is sensitive to its nutritional surroundings and alter its surface-related properties accordingly. Individual root exudates have a significant effect on the specific cell surface properties. For instance, arabinose promotes motility, gluconate promotes EPS formation, and serine increases bacterial cell surface hydrophobicity (panel at **left**). Root exudate exposure leads to diverse surface-related phenotypes, influencing root adherence. As individual bacteria encounter pockets of specific root exudate compounds, they will likely adapt their surface phenotype, e.g., becoming hydrophobic or expressing specific surface sugars. The bacterial phenotypic plasticity points to one mechanism of how plant–microbe interaction is influenced by plant root exudates and that individual RECCs play a specific role at different stages of root attachment.

We tested major phenotypic traits that are known to play roles in root attachment with a goal to understand the role of each in root attachment (Figure 1) (Wheatley and Poole, 2018; Knights et al., 2021). As observed from the results and correlation plot, motility- and chemotaxis-related properties do not play a role in attachment, but only in driving the bacteria to the root. Defects in swimming and swarming did not affect the root attachment capacity of different rhizobial and *Pseudomonas* strains (Albareda et al., 2006). Bacterial cell surface hydrophobicity is an important phenotypic trait that is important for initial attachment but exactly what bacterial components contributed to BCSH is poorly understood. From our results, EPS and biofilm formation ability do not predict initial attachment. Conclusively, the root exudates raffinose and gentisate are good chemoattractants for USDA 110, and, along with arabinose, promote the motility of USDA 110. Serine causes high cell surface hydrophobicity and promotes primary non-specific binding to the root surface. Once attached, more exopolysaccharide production and biofilm formation promoting root exudates, gluconate, and lactate can play a role in the colonization of the root surface (Figure 7).

*B. diazoefficiens* USDA 110 has a genome of 9,105,828 bp (Kaneko et al., 2002). Although several genes related to various symbiotic and nodulation genes have been identified, few studies have been done to identify genes involved in the surface phenotype of USDA 110, other than EPS genes. Our data show that this organism is extremely responsive to its nutritional surroundings and can modify its surface phenotype accordingly (Figure 7). A change in one component of the media can change the whole behavioral and phenotypic structure, and conclusions or observations made using one substrate might not be true for another and may not be true at all for actual soil. In the field, each individual RECC likely plays a significant and specific role to modify the phenotype of the bacterial cell to aid with soybean root attachment at different steps (Figure 7). The results indicate that root exudates influence legume–rhizobia interactions by changing the phenotype of *Bradyrhizobium*. Hence, it is crucial to further understand the basis of bacterial phenotypic plasticity and explore root exudate effects on plant–microbe interaction in soil environments.

## Data availability statement

The raw data supporting the conclusions of this article will be made available by the authors, without undue reservation.

## Author contributions

AS, SS, and VB: conceptualization. AS and VB: methodology. AS and MB: wet lab experiments. AS, MB, and VB: formal analysis. AS: data curation and writing—original draft preparation. AS, MB, SS, and VB: writing—review and editing. All authors contributed to the article and approved the submitted version.
