# Supplementary material for: Bradyrhizobium diazoefficiens USDA 110 displays plasticity in the attachment phenotype when grown in different soybean root exudate compounds
Source: Front Microbiol. 2023 May 18;14:1190396. doi: 10.3389/fmicb.2023.1190396 (PMC10233038; doi:10.3389/fmicb.2023.1190396)
Supplement: Supplementary file 1 [file Data_Sheet_1.docx]

Supplementary Material

**Phenotypic plasticity in attachment and attachment-related surface properties of *Bradyrhizobium diazoefficiens* USDA 110 when grown in different soybean root exudate compounds**

**Armaan Kaur Sandhu^1^, McKenzie Rae Brown^1^, Senthil Subramanian^1,2^ and** **Volker S. Brözel^1,3^***

*** Correspondence:** Corresponding Author: Volker.brozel@sdstate.edu


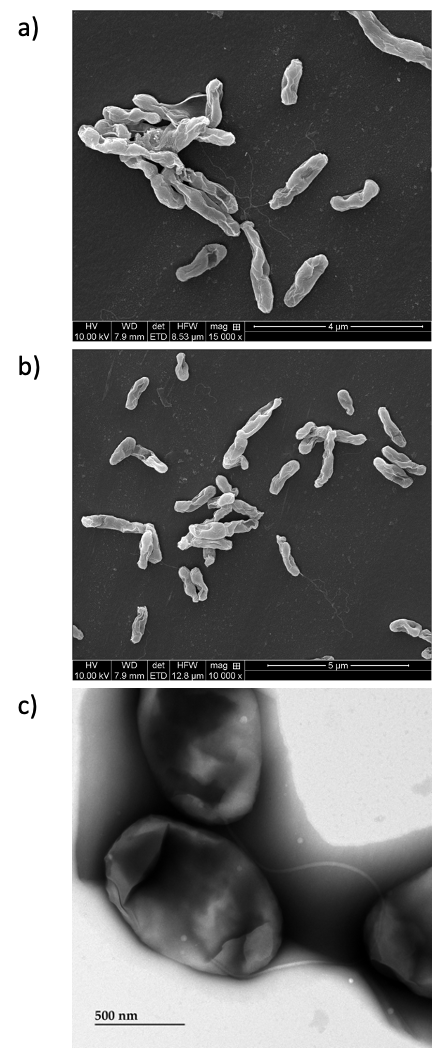


Figure S1: SEM (a, b) and TEM (c) of *B. diazoefficiens* USDA 110 cultured in raffinose, showing sub-polar flagella.


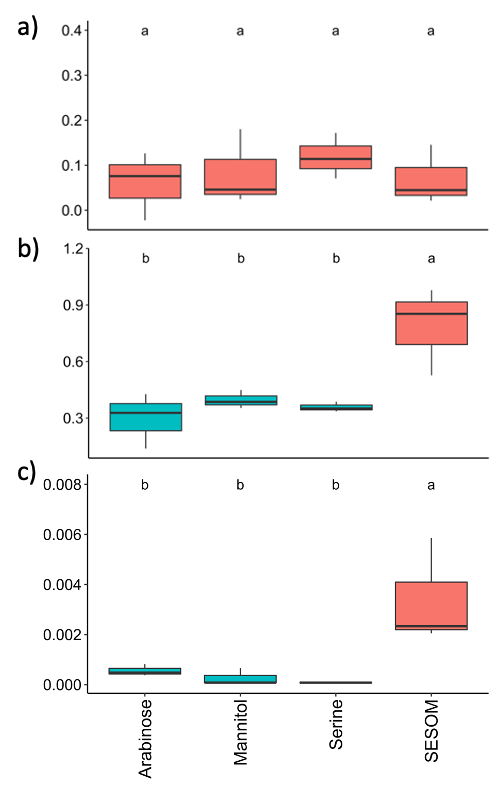


Figure S2: Biofilm formation on polystyrene (a), hydrophobicity determined by the MATH assay using hexadecane (b), and attachment to soybean roots of *B. japonicum* USDA 20 (c).


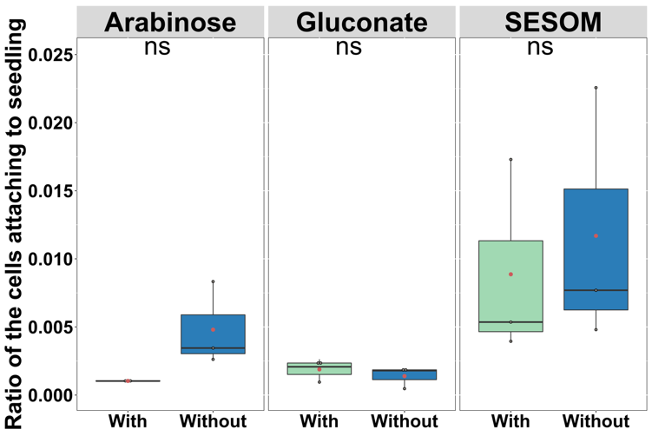


Figure S3: Root attachment of *B. diazoefficiens* USDA 110 cultured in arabinose, gluconate and SESOM before (with, green columns) and after (without, blue columns) removal of EPS.
